# Metabolism of Exogenous D-Beta-Hydroxybutyrate, an Energy Substrate Avidly Consumed by the Heart and Kidney

**DOI:** 10.3389/fnut.2020.00013

**Published:** 2020-02-19

**Authors:** Bernard Cuenoud, Mickaël Hartweg, Jean-Philippe Godin, Etienne Croteau, Mathieu Maltais, Christian-Alexandre Castellano, André C. Carpentier, Stephen C. Cunnane

**Affiliations:** ^1^Nestlé Health Science, Translation Research, Epalinges, Switzerland; ^2^Nestlé Research, Clinical Development Unit, Lausanne, Switzerland; ^3^Nestlé Research, Institute of Food Safety and Analytical Sciences, Lausanne, Switzerland; ^4^Sherbrooke Molecular Imaging Center, Sherbrooke, QC, Canada; ^5^Department of Medicine, Université de Sherbrooke, Sherbrooke, QC, Canada; ^6^Research Center on Aging, Sherbrooke, QC, Canada; ^7^CHUS Research Center, Sherbrooke, QC, Canada; ^8^Department of Pharmacology and Physiology, Université de Sherbrooke, Sherbrooke, QC, Canada

**Keywords:** ketones, acetoacetate, beta-hydroxybutyrate, energy metabolism, medium chain triglyceride, positron emission tomography, nicotinamide adenine dinucleotide

## Abstract

There is growing interest in the metabolism of ketones owing to their reported benefits in neurological and more recently in cardiovascular and renal diseases. As an alternative to a very high fat ketogenic diet, ketones precursors for oral intake are being developed to achieve ketosis without the need for dietary carbohydrate restriction. Here we report that an oral D-beta-hydroxybutyrate (D-BHB) supplement is rapidly absorbed and metabolized in humans and increases blood ketones to millimolar levels. At the same dose, D-BHB is significantly more ketogenic and provides fewer calories than a racemic mixture of BHB or medium chain triglyceride. In a whole body ketone positron emission tomography pilot study, we observed that after D-BHB consumption, the ketone tracer ^11^C-acetoacetate is rapidly metabolized, mostly by the heart and the kidneys. Beyond brain energy rescue, this opens additional opportunities for therapeutic exploration of D-BHB supplements as a “super fuel” in cardiac and chronic kidney diseases.

## Introduction

The ketogenic diet is a very low carbohydrate diet that has shown therapeutic benefits in drug resistant epilepsy (1, 2) and is gaining increased attention in other neurological diseases (3) and in healthy aging (4). Ketones (acetoacetate [AcAc] and D-beta-hydroxybutyrate [D-BHB]) are produced by the liver when blood glucose and insulin decrease (5). Blood ketones above 0.5 mM indicates a ketosis state that reaches 3–5 mM on the ketogenic diet. One of the main benefits of ketones is their ability to act as an alternative energy source to glucose or fatty acids for production of ATP by mitochondria. Caloric restriction and intermittent fasting also produce transient mild-moderate ketosis (6, 7).

**Figure 1:**
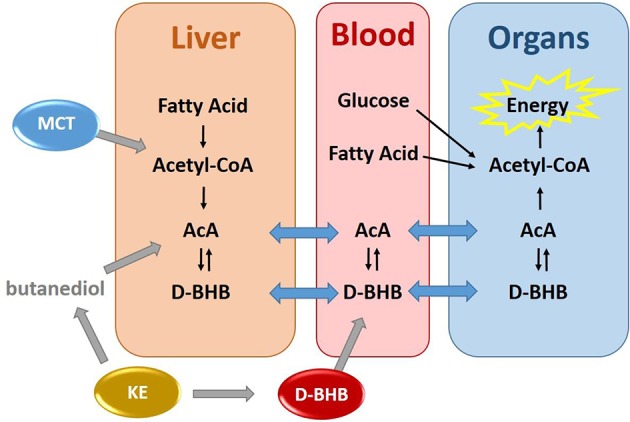
Exogenous production of blood ketones by three ketone precursors–MCT, KE, and D-BHB.

As an alternative to the ketogenic diet, exogenous ketone precursors taken orally achieve mild ketosis in the absence of dietary restriction. They can be grouped in three categories (Figure 1): First, medium chain triglycerides (MCT) composed of a mixture of 8 and 10 carbons fatty acids are efficiently digested to free fatty acids (FFA), directly absorbed, and rapidly metabolized by the liver. The acute production of excess acetyl-CoA drives the production of AcAc and then D-BHB which are both secreted into the systemic circulation (8). While a high dose of MCT can provide a moderate increase in blood ketones (+0.5–1.0 mM), gastrointestinal intolerance and high caloric load limit their use. Second, ketone esters (KE) made of a BHB ester linked to butanediol provide one molecule of D-BHB after digestion, with the butanediol being further metabolized by the liver to D-BHB (9). KE increase blood ketones above 1 mM but are also limited at high dose by their gastric tolerability and severe bitterness (10). Third, perhaps the most physiologic way to raise blood ketones is via the oral intake of D-BHB itself. Exogenous D-BHB is directly absorbed into the circulation, with some of it being converted to AcAc by the liver, and both ketones being distributed throughout the body. Until recently, only racemic mixtures of *dextro* (D) and *levo* (L) BHB (D+L-BHB) were available and oral human studies with them have been reported (9, 11–14). As L-BHB is not metabolized significantly into energy intermediates and is slowly excreted in the urine (9, 15), D+L-BHB would be anticipated to be less ketogenic than pure D-BHB.

Once ketone precursors are absorbed and metabolized, the resulting ketones are taken up by extrahepatic tissues such as brain, heart, muscle, and kidney and metabolized to acetyl-CoA for ATP production in mitochondria (5). Understanding how ketones are utilized by different organs after the intake of a ketone precursor is therefore starting to gain in importance. For instance, brain energy is derived mostly from glucose, but ketones spare brain glucose consumption when they are available (16). When blood ketone levels are increased (either by ketogenic diet or exogenous ketones), the brain utilizes ketones preferentially. Brain ketone metabolism is directly proportional to plasma ketone level over a wide concentration range. An increase in brain ketone metabolism can increase overall brain energy supply in mild cognitive impairment and Alzheimer's disease (17–19). The heart is an energy omnivore and uses both FFA and glucose as major energy substrates (20). Increased blood ketones produced by acute intravenous infusion of D+L-BHB reduces myocardial glucose utilization without affecting myocardial FFA metabolism (21). The kidney uses FFA as its main energy source (22) and, although less well-studied, ketones have been shown to be preferred over FFA, lactate and other endogenous energy substrates for the kidney (23). Despite utilizing ~20% of total body energy intake, the liver cannot use ketones as a source of energy as it lacks the enzyme succinyl-CoA:3-oxoacid-CoA transferase (SCOT) required to convert AcAc back to acetyl-CoA (3). However, the liver contributes to the interconversion of AcAc to D-BHB via mitochondrial D-BHB dehydrogenase (BDH1).

Positron-emission tomography (PET) using the ketone tracer, ^11^C-AcAc, was developed initially to directly observe ketone metabolism in the brain of people developing MCI and AD (24, 25). It has been used to study heart energy metabolism in rodents (26) and has the potential to provide insight into whole body ketone metabolism in humans. Here, the objective was to compare the metabolism of a pure D-BHB oral supplement, i.e., the increase in blood D-BHB and AcAc after D-BHB, to that produced by the ingestion of the same amount of racemic D+L-BHB or MCT. A pilot study was also performed to assess the feasibility of using ^11^C-AcAc PET to observe organ ketone uptake after oral ingestion of D-BHB.

## Materials and Methods

### Test Products

#### D-BHB

14.1 g of pure salts of the D enantiomer (>99% enantiomeric excess) of D-BHB were used. The D-BHB supplement tested was formulated as a mixture of three salts: sodium D-beta-hydroxybutyrate (CAS Registry number 13613-65-5), magnesium (D-beta-hydroxybutyrate)2 (CAS Registry number 586976-57-0), and calcium (D-beta-hydroxybutyrate)2 (CAS Registry number 51899-07-1). Each oral serving provided 12 g D-beta-hydroxybutyric acid, 0.78 g sodium, 0.42 g magnesium, and 0.88 g calcium, citrus flavoring and sweetener (Stevia), dissolved in 150 mL of drinking water.

Chemical purity of beta-hydroxybutyric acid was determined by quantitative ^1^H-nuclear magnetic resonance (NMR). NMR spectra were recorded on a 600 MHz Bruker Avance III spectrometer equipped with a 5 mm TCI cryogenic probe at 300 K using a Topspin 3.5pl7 software (Bruker Biospin).

Enantiomeric purity was determined by chiral high-performance liquid chromatography (HPLC) using an HPLC-UV instrument from Agilent Technologies with a Sumichiral OA6100 (5 μm, 4.6 × 150 mm) column. The mobile phase consisted of 1 mM copper (II) sulfate in water at a flow rate of 1 mL/min. Detection of the peaks was carried out by ultraviolet detection at 254 and 210 nm. Calculation of enantiomeric excess (ee) was expressed in percentage (%) according to the following formula: ee% = [(area of D-BHB– area of L-BHB)/total area of both D and L-BHB combined] × 100.

#### D+L-BHB

14.5 g of an equimolar mixture of commercial D and L beta-hydroxybutyrate salt was used (KetoCaNa, KetoSports, USA). Each serving provided a mixture of 12 g D+L-Beta-hydroxybutyric acid, 1.3 g sodium, 1.2 g calcium, orange flavoring and stevia, dissolved in 150 mL of drinking water.

#### MCT

Fifteen grams of medium chain triglyceride (MCT) (60% caprylic C8 acid and 40% capric C10 acid) emulsified in 70 mL of a 5% aqueous milk protein solution.

#### Standard Breakfast

The meal consisted of 2 boiled eggs, 2 pieces of toast, 1 slice of cheese, and 1 portion of fruit jam, providing a total of 423 kcal (20 g fat, 24 g protein, 32 g carbohydrate). Water was provided *ab-libitum*. The amount of the breakfast was not adjusted to the weight of the participants.

### Pharmacokinetic Study

#### Study Design and Outcomes

The ketogenic potential of D-BHB, D+L-BHB, and MCT was tested in 3 groups of 15 participants. The 3 groups had 11 participants in common and each participant had at least a 5-day washout period between each test product intake.

The groups had a mean age range of 36–38 years, body weight of 72–74 kg, BMI of 23–24 kg/m^2^, fasting plasma ketones of 98–185 μM, fasting plasma glucose of 5.3–5.5 mM, and fasting plasma insulin of 6.1–7.0 mU/L. Detailed demographics for each group are reported in Supplementary Material.

After an overnight fast, participants orally consumed 150 mL of the test product at time 0. At time 30 min, a standard breakfast was provided and consumed over 15 min to mimic the real life situation and explore any interference with the test product. Blood samples (7.5 mL) were taken at regular interval over 4 h [time (min): 0, 15, 30, 45, 60, 120, 180, 240] via a venous catheter. Plasma was analyzed for total ketones and D-BHB using Autokit Total Ketone Bodies and Autokit 3-HB (Wako Diagnostics, Mountain View, CA, USA), respectively. AcAc was then calculated by subtracting total BHB from total ketones. Plasma total BHB (D+L-BHB) was analyzed by ultra-high performance liquid chromatography, tandem mass spectrometry (UHPLC-MSMS; Vantage TSQ, ThermoFischer, Germany), based on the protocol described by Zeng and Cao (27). L-BHB was then calculated by subtracting D-BHB (measured by the enzymatic assay Autokit 3-HB above) from D+L-BHB measured by UHPLC-MSMS. Plasma glucose and insulin were measured using GLUC Flex^®^ reagent cartridges (Siemens Healthcare Diagnostics Inc.) and an ARCHITECT Insulin 8K41 kit (Abbott Laboratories Diagnostics Division, Abbott Park, IL 60064 USA), respectively.

Concentration for maximum effect (C_max_) was calculated as the mean of the maximum concentration reached by each participant. Incremental area-under-the-curve (iAUC) was calculated as the mean of baseline-corrected iAUC for each individual over 4 h. Time for maximum effect (T_max_) was calculated as the mean of T_max_ reached by each individual.

During the 4 h test period, gastro-intestinal tolerability was assessed with a visual analog scale (VAS; 0 to 100) for each of the following symptoms: (1) abdominal discomfort, (2) appetite, (3) gastric reflux, (4) nausea, (5) diarrhea, and (6) headache.

This study was approved by the Ethics Committee of Canton de Vaud (Switzerland) under the generic protocol reference 2018-00503, and all participants provided written informed consent. Procedures were conducted according to the principles of the Declaration of Helsinki. This trial is registered at ClinicalTrials.gov with the identification number NCT03603782.

#### Statistical Analysis

The sample size was based on previous determination of the coefficient of variation of plasma BHB iAUC (SD/mean = 0.0783). With this assumption, in order to detect a 10% difference between the iAUC of two products with a power of 80% and a type 1 error rate of 5%, about 12 participants per group were needed in a complete cross over design frame. Assuming 20% of non-evaluable participants, this resulted in enrollment of 15 participants/group.

To assess a potential carry-over effect, the joint modeling of iAUC and half-life (T_1/2_) was estimated by including product taken, previous product taken, and interaction of product taken and previous product taken as covariates. The covariate-associated coefficients were not different from zero, supporting the assumption that product-related effects were not carried forward over visits.

Exploratory inferential results were obtained with the non-parametric Wilcoxon rank-sum exact test (28). The alpha level was set at 0.05. Significance threshold was not adjusted for multiplicity and samples were considered independent even though some were correlated within participants due to the design of the study (partial cross-over).

### ^11^C-AcAc PET Pilot Study

The full method for the ^11^C-AcAc-PET tracer experiment has been reported previously (16, 17). On the day of the PET scan, prior to injecting the tracer ^11^C-AcAc at 0 min, a 66 year old healthy male (74 kg) fasted for ~6 h then consumed two servings of the D-BHB supplement at −75 min and again at −30 min.

PET images were acquired on a PET/CT (Gemini TF, Philips Healthcare, Eindhoven, the Netherlands). On the contralateral side from the radiotracer injection, blood was arterialized by warming the forearm with a heating pad at 44°C. Blood samples were taken at 3, 6, 8, 12, 20, and 28 min post-injection.

The acquisition protocol was as follows: 370 MBq of ^11^C-AcAc was injected followed by a 10-min dynamic brain acquisition, in list mode, with an isotropic voxel size of 2 mm^3^. Immediately after the dynamic brain acquisition, three whole-body (head to mid-thigh) acquisitions were performed at 18, 25, and 35 min post-injection. The acquisition times per bed position were 30, 45, and 60 s, respectively, for the three scans. Whole body acquisitions were performed with an isotropic voxel size of 4 mm^3^. Finally, an 8 frame per-cycle cardiac-gated acquisition of 15 min was performed 50 min after tracer injection.

PET tracer kinetics were analyzed for the brain (PMOD Technologies Ltd., Zurich, Switzerland). Brain ketone metabolism was assessed with graphical Patlak analysis of ^11^C-AcAc as previously described (16, 17). Briefly, Patlak linearization was used to quantify the brain uptake rate constants of acetoacetate (K_AcAc_) and the cerebral metabolic rate of AcAc and ketones (CMR_AcAc_ and CMR_ketones_). CMR was calculated using the following equation: CMR = K × Cp/LC, where K (min^−1^) is the uptake rate constant, Cp is the arterial plasma concentration, and LC is the lumped constant (1.0 for AcAc) accounting for uptake differences between the tracer and natural molecule. CMR_ketones_ calculation uses the plasma concentrations of AcAc and D-BHB, K_AcAc_, and r_k_ (K_AcAc_/ K_ketones_) set at 1.2 as previously reported (21, 22). CMR_ketones_ = (K_AcAc_x[AcAc]) × (1+(1/r_k_x[AcAc]/[D-BHB])).

To characterize the effect of D-BHB supplementation on ketone uptake in organs besides the brain, ^11^C-AcAc uptake by the liver, kidneys and heart were segmented on the whole-body PET/CT fusion image, and the % dose/g was calculated from the organ volume and injected dose.

Heart reorientation and cardiac function analysis were performed using the cardiac module of PMOD 3.9 to obtain the ventricular volumes, ejection fraction, and polar map (29).

Plasma collected during the PET scan was analyzed for D-BHB and AcAc by automated colorimetric assay on a clinical chemistry analyser (Dimension Xpand Plus; Siemens, Deerfield, IL, USA) as previously described (8).

This PET study was approved by the CIUSSS de l'Estrie–CHUS Research Ethics Committee.

## Results

### Pharmacokinetics Study

**Figure 2:**
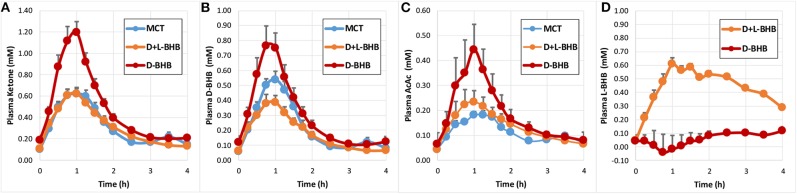
Blood ketone kinetics following gram-matched oral doses of D-BHB, D+L-BHB, and MCT in 15 fasted participants at rest. **(A)** Plasma ketone, **(B)** D-BHB, **(C)** AcAc, and **(D)** L-BHB levels over 4 h. Values are means + SEM.

**Figure 3:**
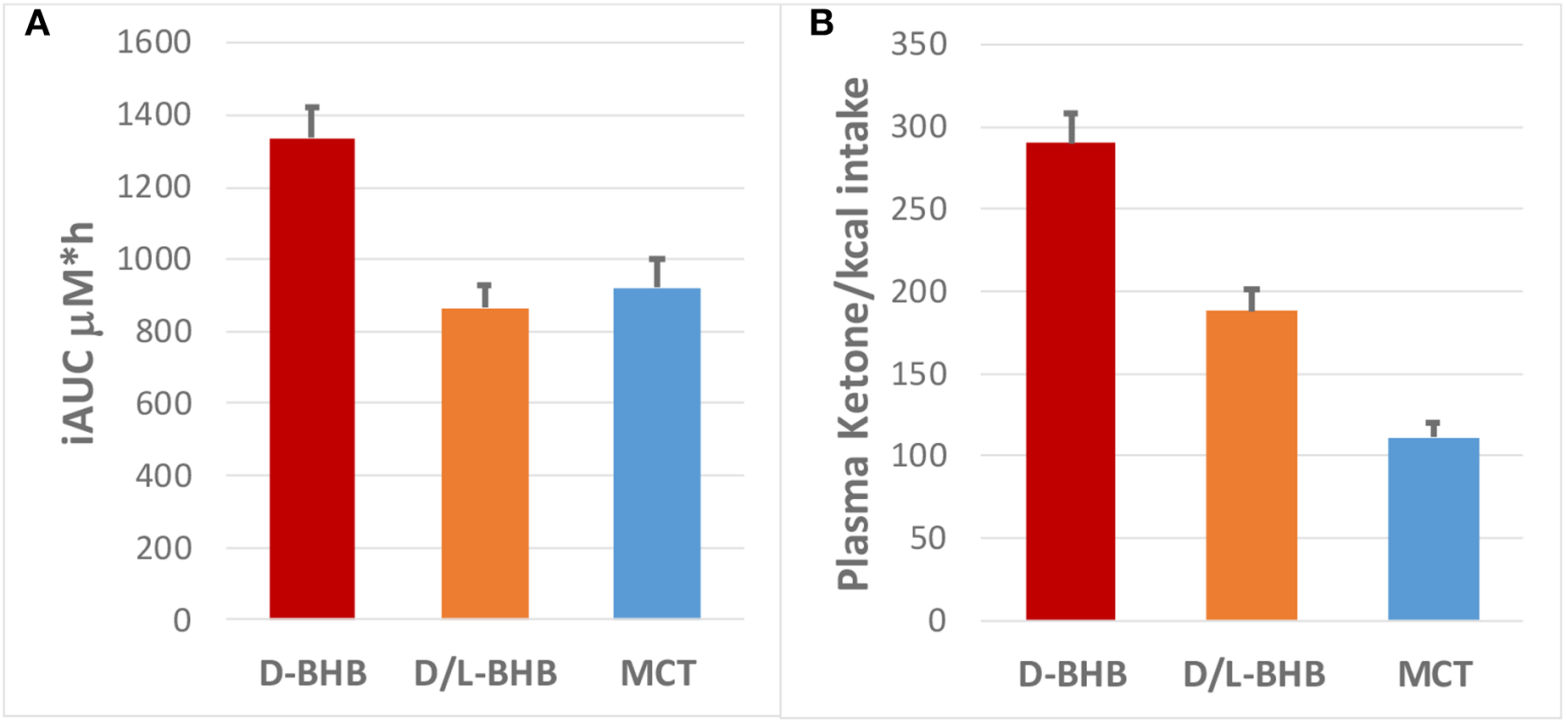
**(A)** Plasma ketone iAUC (μM^*^h) for D-BHB, D+L BHB and MCT, and **(B)** their respective ketone production per calorie consumed (iAUC/kcal).

Following intake of the D-BHB, blood ketones rapidly increased (Figure 2A, Supplementary Table 2) to a C_max_ of 1.2 ± 0.1 mM. When the same amount of D+L-BHB or MCT was consumed, a ~50% lower C_max_ from baseline was observed compared to D-BHB (C_max_ D+L-BHB 0.62 ± 0.05 mM and MCT 0.62 ± 0.06 mM; *p* < 0.001 vs. D-BHB for both). T_max_ was reached in about 1 h for each product (Supplementary Table 3), and ketone levels came back to baseline values after 3–4 h. The iAUC for D-BHB was ~1.5 fold higher than for D+L-BHB or MCT (Figure 3A, Supplementary Table 1; *p* < 0.005 for both).

Analysis of blood D-BHB and AcAc revealed a similar pattern for the three products (Figures 2B,C). However, MCT produced proportionally less blood AcAc and more blood D-BHB than oral D-BHB or D+L-BHB. The calculated ratio [mean AcAc iAUC/mean D-BHB iAUC] was 0.44 for MCT, a value ~30–40 % lower than for D-BHB (0.63) and D+L-BHB (0.76) (Supplementary Table 1; D-BHB vs. MCT *p* = 0.0102; D+L-BHB vs. MCT *p* < 0.0001; D+L-BHB vs. D-BHB *p* = 0.173). The baseline ratio AcAc/D-BHB was ~0.7, suggesting that MCT lowered the AcAc/D-BHB blood ratio while producing ketones. Similar ratios were obtained when using values derived from C_max_ [mean AcAc C_max_/mean D-BHB C_max_] (Supplementary Table 2).

As expected, oral intake of D+L-BHB resulted in a significant increase in plasma L-BHB over the first hour (Figure 2D), follow by a slow decrease over the next 3 h, without coming back to baseline. In comparison, oral intake of D-BHB did not produce an increase in plasma L-BHB.

Ketone production per calorie ingested [MCT: 8.3 kcal/g; BHB: 4.6 kcal/g (42)] was significantly higher for D-BHB than for D+L-BHB and MCT (Figure 3B, *p* < 0.0001 for all three comparisons D-BHB vs. D+L BHB vs. MCT). Increased glucose and insulin levels as a result of the intake of the meal 30 min after each test product was consumed were not significantly different across the three groups (Supplementary Table 4 and Supplementary Figure 2). Tolerability of the three products, evaluated by a GI visual assessment score, was similar with no clinically significant differences (Supplementary Figure 1). Amongst some of the GI effect self-reported, decreased appetite and some diarrhea were the most common for the three products.

### ^11^C-AcAc PET Pilot Study

**Figure 4:**
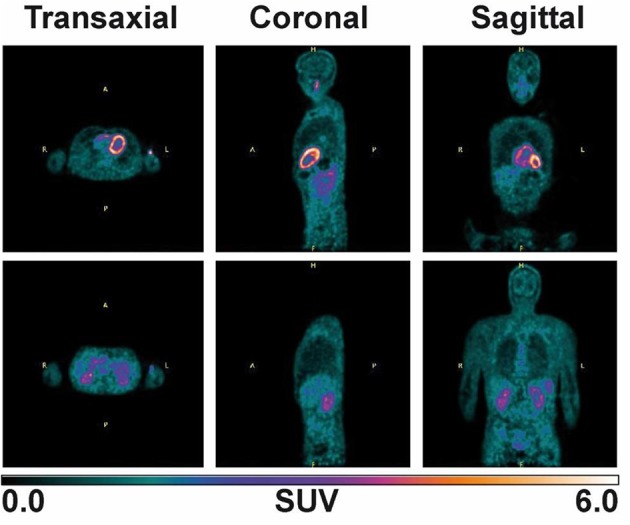
Whole body image after supplementation with two 15 g D-BHB doses, one at −75 min and the other at −30 min prior ^11^C-AcAc infusion (330 MBq). An 8 min scan (30 s per bed) was acquired 18 min post-injection of the radiotracer.

**Figure 5:**
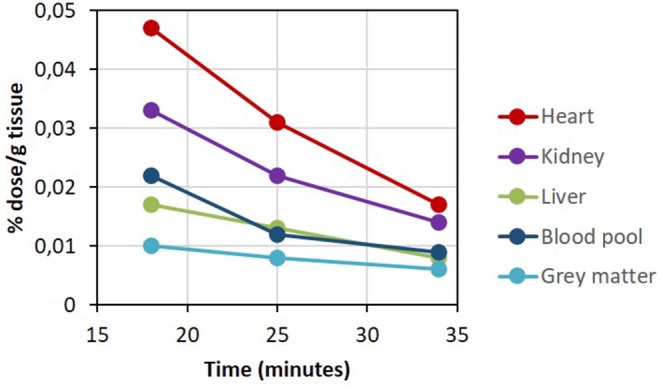
^11^C-AcAc organ distribution after D-BHB oral intake.

Plasma ketone levels were between 0.9 and 1.2 mM during the first 30 min of the PET scan acquisition. The first whole-body PET/CT biodistribution of ^11^C-AcAc 18 min post injection of the radiotracer is illustrated in Figure 4. The radiotracer was rapidly taken up mainly by the heart, where both ventricles and the atriums could be observed. ^11^C-AcAc uptake estimates per gram of tissue over three time points are summarized in Figure 5. Excretion of the radiotracer was observed in the urinary bladder and the salivary glands.

From the dynamic brain scan, CMR_AcAc_ and K_AcAc_ could be determined for all main regions of the brain (Supplementary Table 5) and compared to baseline values previously determined in healthy young adults (16). Overall and compared to baseline, each region demonstrated an increase in CMR_AcAc_ and K_AcAc_ of ~4.7 and 2.3-fold, respectively, about 1 h after taking D-BHB. This indicated that AcAc is effectively taken by the brain and by other organs particularly the heart and the kidney.

**Figure 6:**
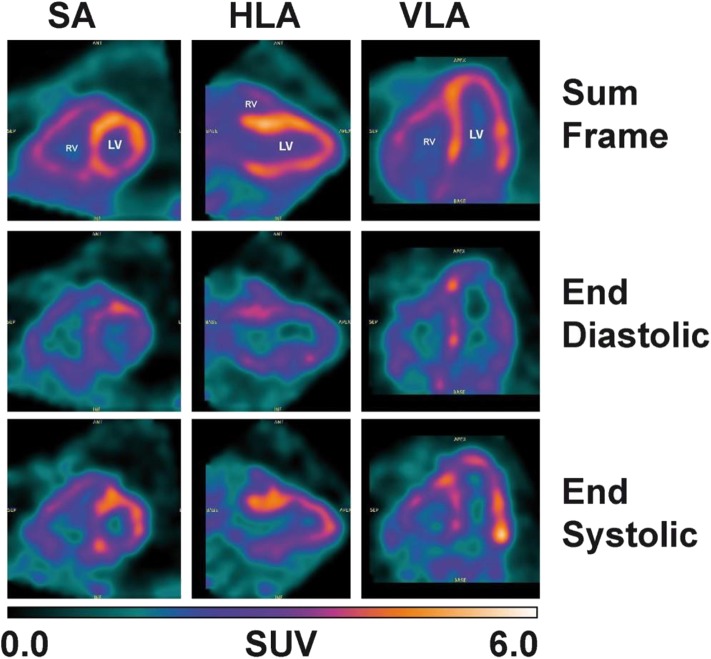
Heart image: 15 min cardiac-gated acquisition (8 frames) obtained 50 min post-^11^C-AcAc injection. Left ventricle (LV), right ventricle (RV), horizontal long axis (HLA), vertical long axis (VLA) and short axis (SA) images.

Despite the fact that a dynamic cardiac scan could not performed in the present study, assessment of cardiac function was still possible with the gated heart PET image (Figure 6). The values of the end-diastolic and end-systolic volumes as well as the ejection fraction of the left ventricle calculated from the ^11^C-AcAc metabolism were 100, 47 ml, and 52%, respectively.

## Discussion

To our knowledge, this is the first report of the metabolism of D-BHB in humans and its link to the organ distribution of the ketone, AcAc, by PET imaging. This study shows that D-BHB is rapidly absorbed and metabolized (Figure 2). It also increases blood ketones above 1 mM, a level ~1.7 fold greater than the same dose of D+L-BHB or MCT, and this despite the lower caloric load of D-BHB. Moreover, the results of the pilot whole body ^11^C-AcAc-PET experiment support the feasibility of this technique to measure organ-specific exogenous ketone utilization and they suggest that exogenous ketones are very actively utilized by the heart and kidney.

Ketone production from an exogenous dietary source has been traditionally achieved by MCT. This requires a bolus intake to saturate the liver with MCFA, producing excess acetyl-CoA which is then transformed to AcAc and BHB, which are released into systemic circulation. The C_max_ achieved with MCT is usually between 300 and 600 μM, with higher values being difficult to reach due to GI side effects and liver saturation. Here we show that D-BHB, a natural and biologically active ketone isomer, raises blood ketone C_max_ above 1 mM without noticeable side effects. In comparison, an equivalent dose of D+L-BHB or MCT only achieved half this ketone level, with similar T_max_ at 1 h. Thus, compared to D+L-BHB, D-BHB significantly reduces the salt intake needed to achieve the same plasma ketone response.

Results from a previous study (9) comparing KE to D+L-BHB showed that at the same dose of D-BHB equivalent, the increase blood ketone iAUC had the same magnitude, suggesting that exogenous D-BHB and KE produce similar ketosis. We note that the T_max_ of D+L-BHB was significantly longer (~90 min) compared to KE (~20 min) at the same dose, suggesting that D-BHB could have a longer duration of action than KE. We also found that D+L-BHB resulted in a significant increase of plasma L-BHB that eliminate slowly, consistent with a previous report (9) and indicating a difference in the metabolic utilization of D and L-BHB. While D-BHB is readily utilized as an energy source, the L-isoform is eliminated slowly in the urine and does not contribute significantly to the biological activity of ketone precursors as an energy source. Finally, D-BHB provided higher ketones per calorie consumed than MCT: the caloric density of D-BHB (4.6 kcal/g) is half that of MCT (8.2 kcal/g), making D-BHB a 2.6 fold more energetically efficient ketone precursor vs. MCT (based on iAUC over 4 h).

The present study also revealed that D-BHB conversion to AcAc provided a ~43% higher AcAc/D-BHB blood ratio (0.63) than the same dose of MCT (0.44). In this respect, KE seems to behave more like MCT, with a ratio around 0.2–0.4 (3), while on the ketogenic diet, this ratio remains unchanged around 0.5 (16). Since D-BHB needs to be converted to AcAc by BDH-1 within each organ before being metabolized to acetyl-CoA (3), a higher AcAc/D-BHB ratio could be energetically more effective. Moreover, as this conversion requires the co-factor, nicotinamide adenine dinucleotide (NAD^+^), to be reduced to NADH, a higher AcAc/D-BHB might better preserve the mitochondrial NAD^+^/NADH ratio. Interestingly, a higher NAD^+^/NADH ratio has been associated to better health (30, 31), and can be increased in the brain by nutritional ketosis (32, 33), suggesting that D-BHB might be superior to MCT or KE in this respect, a point meriting further investigation.

PET has been invaluable in measuring energy metabolism at the organ level in humans. For example, with ^18^F-FDG, it clearly shows that glucose is the main energy substrate of the brain and that a fatty acid such as ^11^C-palmitate is preferentially utilized by the heart and the liver (34, 35). Our pilot ^11^C-AcAc-PET experiment aimed simply to demonstrate the feasibility of measuring ketone uptake by the main organs when exogenous D-BHB is administered orally to humans. The key advantage of ^11^C-AcAc as a metabolic radiotracer is that it equilibrates rapidly with D-BHB in blood and is indistinguishable from endogenous AcAc, so reflects the actual metabolism of ketones. The whole-body scan revealed for the first time that the heart and kidney are major contributors to the metabolism of exogenous AcAc after ingestion of a D-BHB supplement.

Most of the heart's energy requirement is normally provided by fatty acids and glucose (20). Our results confirm that the heart has the capacity to utilize other substrates such as ketones (21) and that ketone metabolism appears to be linked to the metabolism and clearance of the radiotracer by the myocardium. On a per gram basis, the kidney consumes a large amount of energy mostly derived from fatty acids, but our data show that ketones can clearly also be used effectively for mitochondrial respiration in the kidney as well.

The cerebral metabolic rate for ketones (CMR_ketones_) obtained here with D-BHB (3.0 μmol/100 g/min) exceeded that obtained after MCT supplementation in mild cognitive impairment [2.49 μmol/100 g/min; (17)] and Alzheimer's disease [1.9–2.0 μmol/100 g/min; (18)]. While the CMR values obtained here for D-BHB are preliminary, our data suggest that a D-BHB supplement represents a promising intervention to provide ketones that can rescue the brain energy glucose deficit in these conditions (17, 25).

^11^C-AcAc uptake by the liver (Figures 4, 5) could reflect both the conversion of ^11^C-AcAc to ^11^C-D-BHB by BHD-1 and the high blood reservoir capacity of the liver, but do not reflect ketone metabolism by the liver itself. We show here that the blood radioactivity values of ^11^C-AcAc were similar to those of the liver (see Figure 5) supporting the view that the liver produces but does not metabolize ketones (5). This is consistent with the liver radioactivity after ^11^C-AcAc infusion being due to blood and not to the conversion of AcAc into AcAc-CoA and other energy intermediates (5).

Recent studies have shown the potential importance of ketones in cardio-metabolic health. Infusion of D+L-BHB has beneficial hemodynamic effects in adult patients with heart failure and lower ejection fraction (36). In children with fatty acid oxidation defects such as multiple acyl-CoA dehydrogenase deficiency, D+L-BHB improved heart function and cognitive performance, which resulted in better walking ability and correction of neurological symptoms (37). Moreover, clinical trials in diabetes with sodium/glucose co-transporter-2 (SGLT-2) inhibitors, which increase plasma ketones (38), show markedly reduced risk of cardiovascular events and kidney failure (39). Given the significant dysregulation of energy metabolism in type 2 diabetes, it is plausible that part of the effect of SGLT2 inhibitors on heart and kidney is mediated via improved ATP production using ketones as an alternative energy fuel to fatty acid and glucose (38, 40). This opens additional opportunities for therapeutic exploration of D-BHB supplements beyond energy rescue of the brain.

This study has several limitations. First, the pharmacokinetic comparison of D-BHB, D+L-BHB and MCT was an acute, single 4 h study; the extent to which it reflects long-term differences in their metabolism remains to be determined. Second, the whole-body PET scan was done on a single person and (except for brain) was a semi-quantitative comparison across organs. The brain was chosen for the quantitative scan (CMR_AcAc_). Dynamic PET scanning is required in order to quantify the tracer uptake and cannot be done simultaneously on the brain as well as other organs. Cardiac gated image quality could be improved if the image was acquired sooner after tracer injection. Third, ketone production and metabolism vary depending on post-prandial metabolic status, which possibly influences organ ketone distribution. Hence, follow-up of this single observation under different feeding conditions will be needed to verify the relative differences across organs.

## Conclusions

D-BHB appears to be a promising supplement to produce significantly higher blood ketones than D+L-BHB or MCT, and at a lower calorie intake for an equivalent dose. Moreover, exogenous D-BHB does not appear to lower the blood AcAc/D-BHB ratio, which, altogether, might make it a more effective “super fuel” compared to other ketone precursors such as MCT, D+L-BHB or KE.

The pilot ^11^C-AcAc-PET study clearly identifies the heart and kidney as significant consumers of exogenous ketones, in fact, more than the brain. Therefore, D-BHB supplementation could be tested in conditions such as heart failure and diabetic cardiomyopathy to improve cardiac energy efficiency and function, and in chronic kidney disease.

## Data Availability Statement

All datasets generated for this study are included in the article/Supplementary Material.

## Ethics Statement

The studies involving human participants were reviewed and approved by the Ethics Committee of Canton de Vaud (Switzerland) under the generic protocol reference 2018-00503, Trial registration number NCT03603782 (pharmacokinetic study), and by the CIUSSS de l'Estrie, CHUS Research Ethics Committee, Sherbrooke (Canada; PET study). The patients/participants provided their written informed consent to participate in this study. Written informed consent was obtained from the individual(s) for the publication of any potentially identifiable images or data included in this article.

## Author Contributions

MH, EC, C-AC, SC, and BC designed the studies. Data collection was performed by MH, EC, C-AC, J-PG, and MH. C-AC and EC analyzed data. The manuscript was drafted by BC and SC and all authors discussed the results and revised critically the manuscript.

### Conflict of Interest

BC, MH, and J-PG are employees of Nestlé. SC has consulted for Nestlé, Bulletproof and Accera, and received research funding and/or research materials from the Alzheimer Association (USA), Mitacs, FRQS, Abitec and Nestlé. AC declares research funding from CIHR, Canadian Diabetes Association, Fonds de recherche du Quebec–Santé, Janssen, Merck, UniQure, Caprion, Eli Lilly, Novo Nordisk, GlaxoSmithKline, Novartis, Pfizer, Philips, Sanofi, Siemens, and Amgen and consulting/advisory panel participation or conference fees from Merck, Amgen, Janssen, UniQure, Servier, Novo Nordisk, and Novartis. EC, MM, and C-AC declare no competing financial interests.
