# Supplementary material for: Metabolism of Exogenous D-Beta-Hydroxybutyrate, an Energy Substrate Avidly Consumed by the Heart and Kidney
Source: Front Nutr. 2020 Feb 19;7:13. doi: 10.3389/fnut.2020.00013 (PMC7042179; doi:10.3389/fnut.2020.00013)
Supplement: Supplementary file 1 [file Data_Sheet_1.docx]

Supplementary Material

# Subject demographics

In the first group, fifteen healthy individuals tested the MCT supplement (37.5 ± 5.6 y, body weight 72.1 ± 11.6 kg, BMI 24.0 ± 2.3 kg/m^2^, fasting plasma ketones 98.6 ± 73.6 μM, fasting plasma glucose 5.3 ± 0.4 mM, fasting plasma insulin 6.1 ± 2.8 mU/L, 5 females and 10 males).

In the second group, fifteen healthy individuals tested the D-BHB (age 36.1 ± 7.6 years, body weight 73.6 ± 7.3 kg, BMI 23.9 ± 1.3 kg/m2, fasting plasma ketones 185.5 ± 173.7 μM, fasting plasma glucose 5.3 ± 0.4 mM, fasting plasma insulin 6.1 ± 3.1 mU/L, 4 females and 11 males).

In the third group, fifteen healthy individuals tested the D+L-BHB (age 34.6 ± 6.2 years, body weight 71.7 ± 7.7 kg, BMI 23.8 ± 1.7 kg/m2, fasting plasma ketones 107.3 ± 68.7 μM, fasting plasma glucose 5.4 ± 0.4 mM, fasting plasma insulin 6.6 ± 2.8 mU/L, 5 females and 10 males).

The groups had 11 participants in common, with a minimum washout time of 5 days between tests.

# Supplementary Figures and Tables

**Supplementary Table S1:** Incremental Area Under the Curve iAUC (0-4 h) for plasma ketones (D-BHB+AcAc), D-BHB and AcAc (in μM*h), and AcAc/D-BHB ratio (n=15).

|  | **iAUC ketones** | SE | **iAUC D-BHB** | SE | **iAUC AcAc** | SE | **AcAc/ D-BHB** |
| --- | --- | --- | --- | --- | --- | --- | --- |
| **D-BHB** | 1337 | 83 | 824 | 55 | 520 | 41 | 0,63 |
| **D+L-BHB** | 865 | 62 | 497 | 34 | 375 | 32 | 0,76 |
| **MCT** | 922 | 78 | 679 | 59 | 296 | 28 | 0,44 |

**Supplementary Table S2:** C_max_ (mM) for plasma ketones (D-BHB+AcAc), D-BHB, AcAc and AcAc/D-BHB (n=15).

|  | **C_max_ ketones** | SE | **C_max_ D-BHB** | SE | **C_max_  AcAc** | SE | **AcAc/ D-BHB** |
| --- | --- | --- | --- | --- | --- | --- | --- |
| **D-BHB** | 1.20 | 0.10 | 0.75 | 0.06 | 0.44 | 0.05 | 0.59 |
| **D+L-BHB** | 0.62 | 0.05 | 0.39 | 0.03 | 0.23 | 0.02 | 0.60 |
| **MCT** | 0.62 | 0.06 | 0.54 | 0.06 | 0.18 | 0.02 | 0.34 |

**Supplementary Table S3:** T_max_ (hour) for plasma ketones (D-BHB+AcAc), D-BHB, and AcAc (n=15).

|  | **T_max_ ketones** | SE | **T_max_  D-BHB** | SE | **T_max_ AcAc** | SE |
| --- | --- | --- | --- | --- | --- | --- |
| **D-BHB** | 0.9 | 0.03 | 0.9 | 0.03 | 1.0 | 0.04 |
| **D+L-BHB** | 0.9 | 0.05 | 0.9 | 0.09 | 1.2 | 0.11 |
| **MCT** | 1.0 | 0.08 | 1.1 | 0.06 | 1.1 | 0.09 |

**Supplementary Table S4:** Incremental Area Under the Curve iAUC (0-4 h) for plasma glucose (mM*h) and insulin (mU/L*h) (n=15).

|  | **iAUC Glucose** | SE | **iAUC Insulin** | SE |
| --- | --- | --- | --- | --- |
| **D-BHB** | 1.60 | 0.33 | 53.5 | 8.9 |
| **D+L-BHB** | 1.30 | 0.24 | 52.6 | 12.3 |
| **MCT** | 1.50 | 0.20 | 54.0 | 5.9 |

**Supplementary Table S5:** Cerebral metabolic rate (CMR) of acetoacetate (AcAc) and ketones (AcAc + D-BHB) 10 min after the tracer injection (n=1).

|  | **Baseline (ref 16)** | **D-BHB** | **Baseline (ref 16)** | **D-BHB** |
| --- | --- | --- | --- | --- |
| Region | **CMR_AcAc_** μmol/100 g/min | **CMR_AcAc_**  μmol/100 g/min | **K_AcAc_** (min^-1^) | **K_AcAc_**  (min^-1^) |
| White-matter | 0,17 | 0,73 | 0,010 | 0,022 |
| Cerebellum | 0,20 | 1,02 | 0,012 | 0,031 |
| Subcortical | 0.16 | 0,67 | 0,010 | 0,020 |
| Frontal | 0.28 | 1,32 | 0,018 | 0,039 |
| Occipital | 0.31 | 1,37 | 0,019 | 0,041 |
| Temporal | 0.26 | 1,29 | 0,017 | 0,039 |
| Parietal | 0.30 | 1,43 | 0,019 | 0,043 |
| Cingulate | 0.22 | 1,05 | 0,014 | 0,031 |
| ctx-average | 0.27 | 1,29 | 0,017 | 0,039 |
| Whole Brain | 0.25 | 1,16 | 0,016 | 0,035 |
|  |  |  |  |  |
|  | CMR_ketones_ | CMR_ketones_ |  |  |
| Whole Brain | 1,17 | 2,98 |  |  |

1. Note: baseline cerebral metabolic rate for AcAc determined by arterio-venous measurement reported a whole brain CMR_AcAc_ of 0.7 (ref: Brain metabolism during short-term starvation in humans. Hasselbalch SG, Knudsen GM, Jakobsen J, Hageman LP, Holm S, Paulson OB. J Cereb Blood Flow Metab. 1994 Jan;14(1):125-31. PMID: 8263048)

**Supplementary Figure S1:** Gastrointestinal tolerability evaluation using a Visual Assessment Score – mean + SEM (Scale 0-100; n=15/test).

**Supplementary Figure S2:** Incremental mean plasma glucose and insulin concentration over time (n=15).
